# The impact of race and ethnicity in breast cancer—disparities and implications for precision oncology

**DOI:** 10.1186/s12916-022-02260-0

**Published:** 2022-02-11

**Authors:** Kelly A. Hirko, Gabrielle Rocque, Erica Reasor, Ammanuel Taye, Alex Daly, Ramsey I. Cutress, Ellen R. Copson, Dae-Won Lee, Kyung-Hun Lee, Seock-Ah Im, Yeon Hee Park

**Affiliations:** 1 Department of Epidemiology and Biostatistics, College of Human Medicine, Michigan State University, East Lansing, MI 48824 USA; 2 Department of Internal Medicine, Division of Hematology Oncology, O’Neal Comprehensive Cancer Center, University of Alabama at Birmingham, Birmingham, AL USA; 3 Cancer Sciences Academic Unit, Faculty of Medicine, University of Southampton and University Hospital Southampton, Southampton, SO16 6YD UK; 4 Department of Internal Medicine, Seoul National University Hospital, Seoul National University College of Medicine, Seoul, Republic of Korea; 5 Cancer Research Institute, Seoul National University College of Medicine, Seoul, Republic of Korea; 6 Division of Hematology-Oncology, Department of Medicine, Samsung Medical Center, Sungkyunkwan University School of Medicine, 81 Irwon-ro Gangnam-gu, Seoul, 06351 Korea

**Keywords:** Breast cancer, Race, Ethnicity, Disparities, Clinical outcomes, Socioeconomic status, CDK4/6 inhibitors, PARP inhibitors, BRCA mutations, Targeted therapy, Precision oncology

## Abstract

Breast cancer is the most commonly diagnosed cancer worldwide and is one of the leading causes of cancer death. The incidence, pathological features, and clinical outcomes in breast cancer differ by geographical distribution and across racial and ethnic populations. Importantly, racial and ethnic diversity in breast cancer clinical trials is lacking, with both Blacks and Hispanics underrepresented. In this forum article, breast cancer researchers from across the globe discuss the factors contributing to racial and ethnic breast cancer disparities and highlight specific implications of precision oncology approaches for equitable provision of breast cancer care to improve outcomes and address disparities.

## Introduction

### Kelly Hirko

Female breast cancer is the most commonly diagnosed cancer worldwide, with an estimated 2.3 million new breast cancer cases in 2020, representing nearly 12% of all cancer diagnoses and 7% of all cancer deaths [1]. Breast cancer incidence and mortality rates vary widely across geographic regions and racial and ethnic populations. For example, breast cancer incidence rates are higher, while mortality rates are lower, in transitioned versus transitioning countries [1]. Likewise, African-American women have a lower incidence of breast cancer compared to White women, but a higher overall mortality [2]. Breast cancer incidence and mortality also vary across Asian [3] and Hispanic/Latino [4] populations. Overall, breast cancer survival has increased over the past several decades, yet substantial geographic and racial and ethnic disparities in clinical outcomes persist.

Observed breast cancer disparities are largely driven by social determinants of health, including access to screening and quality cancer care [5], as well as differences in the risk factors [5] and comorbidity burden, occurring largely as a result of socioeconomic inequalities [6–8]. Indeed, comprehensive cancer treatment is reportedly available in more than 90% of high-income countries, but less than 15% of low-income countries [9]. While race and ethnicity are highly correlated with socioeconomic status (SES), racial and ethnic disparities in breast cancer risk and outcomes are reduced but not eliminated after adjusting for SES [10, 11]. Increased efforts to identify novel strategies for prevention and early detection and to equitably implement and translate findings into practice are urgently needed to address breast cancer disparities.

Precision oncology approaches and targeted therapies hold tremendous potential to improve breast cancer outcomes but are based largely on biological mechanisms and genetics that are not well-studied in minorities [12]. Indeed, racial and ethnic diversity in breast cancer clinical trials is lacking, with both Black and Hispanics vastly underrepresented [12]. This lack of diversity in trials to test targeted therapies has important implications for breast cancer disparities given the marked variation in the prevalence of certain mutations of high-penetrance genes, such as BRCA1 and BRCA2 [13], and breast tumor subtypes across racial and ethnic populations [14–16]. For example, Black women have a lower frequency of PIK3A mutations than White women [17] and are therefore less likely to benefit from targeted therapies with PI3K inhibitors. Moreover, utilization of targeted therapies has been disproportionate across racial and ethnic minority populations and varies according to socioeconomic status [18]. Access to targeted therapies requires additional testing to determine the eligibility based on the presence of specific mutations, adding complexity and cost, which may disproportionately impact underresourced populations. Inclusion of globally diverse populations in breast cancer clinical trials is imperative to develop therapies that have the potential for a broad reach and to achieve more equitable clinical outcomes.

In this forum article, breast cancer researchers from across the globe discuss factors contributing to observed racial and ethnic breast cancer disparities and highlight specific challenges and implications of precision oncology approaches for equitable provision of breast cancer care. Importantly, race and ethnicity are social constructs, without scientific or biological meaning [19, 20]. As such, the findings from research on racial and ethnic disparities presented in this forum consider sociodemographic factors and social determinants, including structural racism, and use race and ethnicity as a lens to investigate breast cancer inequities [19–22].

- In the first section, Drs. Gabrielle Rocque, Erica Reasor, and Ammanuel Taye discuss the disparities in breast cancer among African-American and Hispanic-American women. Here, researchers illustrate how the experience of systemic racism and allostatic load, as well as differences in the distribution of breast cancer molecular subtypes and tumor genomic signatures of breast tumors, contribute to observed racial and ethnic breast cancer disparities in the USA. The authors advocate for increasing racial and ethnic diversity in clinical trials to ensure that targeted therapies do not contribute to widening breast cancer disparities.
- Drawing largely on the findings from a prospective cohort of young-onset breast cancer in the UK, the authors Drs. Alex Daly, Ramsey Cutress, and Ellen Copson in the next section of this forum article describe observed racial differences in pathological and clinical characteristics of young-onset breast cancer (diagnosed before age 50 years) and potential determinants of delayed breast cancer presentation among racial and ethnic minority women. Furthermore, racial and ethnic differences in the treatment management in young-onset breast cancer are discussed. The authors express the importance of community-engaged research to develop appropriate and acceptable solutions to mitigate disparities.
- Next, Drs. Dae-Wun Lee, Kyung-Hun Lee, and Professor Seock-Ah Im provide an important perspective on the impact of ethnicity on the efficacy and toxicity of cyclin-dependent kinase inhibitors in breast cancer, focusing on Asian women. Here, the authors describe the molecular differences in breast cancer between Asian and Western women and discuss the underrepresentation of Asian women in endocrine therapy trials for breast cancer.
- In the final section of this forum, Professor Yeon Hee Park focuses the discussion on ethnic differences in BRCA mutations and the use of PARP inhibitors in hereditary breast cancer. Clinical and genomic analyses of germline BRCA mutations and somatic TP53 mutations among Korean women with breast cancer are described and implications for utilization of PARP1 inhibitors are discussed.

In summary, this forum article is meant to stimulate additional research to mitigate persistent racial and ethnic disparities in breast cancer and ensure that precision oncology approaches and targeted therapies do not exacerbate existing inequities.

### Competing interests

KH declares that there are no competing interests.

## Breast cancer disparities in the USA

Gabrielle Rocque, Erica Stringer-Reasor, Ammanuel Taye

### Introduction

One in ten women will be diagnosed with breast cancer worldwide [23]. Cancer mortality for the > 250K women in the United States of America (USA) with breast cancer has improved as diagnostic testing and drug therapies have evolved [24]. However, these improvements have not been equally realized among all patients. Breast cancer survival rates vary greatly by geographic region, with approximately 80% in North America, 60% in middle-income countries such as Japan and Sweden, and below 40% in low-income underdeveloped countries [23]. Additionally, outcomes vary among racial and ethnic minority populations, as well as other underserved populations, both of which experience health disparities. For example, an estimated 3.1% of all Black women will ultimately die from breast cancer compared to 2.6% of White women [25]. Even after adjusting for age, the mortality rate for Black women (28.4 per 100,000 women) exceeds that of White, Hispanic, and Asian women (20.3, 14.0, 11.5 per 100,000, respectively) [25]. These reasons for breast cancer disparities in the USA are complex. They result from the interplay between social determinants of health, allostatic load, tumor biology, and access to high-quality cancer care, including clinical trial opportunities [26]. Careful consideration of these factors will be critical to overcoming disparities and improving precision medicine for all individuals.

### Structural barriers to health equity in the USA

Institutional racism, also known as systemic racism, results in unequal wealth distribution, lack of employment opportunities, inequitable education, legal injustices, few leadership roles occupied by Black, Indigenous, and People of Color (BIPOC) persons, and low funding and staffing at safety net hospitals which leads to inadequate access to healthcare for segments of the population [26, 27]. In the USA, the COVID-19 pandemic has further widened these disparities [26]. Access to quality health care remains a challenge and begins even before a breast cancer diagnosis, at the time of screening. Mammography screening leads to the detection of smaller, more curable cancers. Black women in urban areas and Hispanic women in both urban and non-urban areas are more likely to report barriers to mammography than non-Hispanic White women [28]. These barriers likely play in the stage distribution of breast cancer. Despite the similar or greater incidence of breast cancer, non-Hispanic White women have the highest rates of early-stage, localized disease at 66%, with Black, Hispanic, and Asian/Pacific Islander patients having lower localized disease rates of 56%, 58%, and 64%, respectively [25]. Furthermore, after diagnosis, the differences among surgery, radiation, and medical therapies, as well as treatment delays, exacerbate these screening challenges [29]. In a cross-sectional study of women with early-stage breast cancer, underuse of appropriate adjuvant therapy was observed in 34% of Black women and 23% of Hispanic women, but only 16% of White women [30]. In addition, non-Hispanic White patients are significantly more likely to receive adjuvant radiation (OR 1.48) than Black patients when recommended [31]. Access to genetic testing also plays a role in the treatment and prevention of breast cancer. A recent publication by Hu et al. evaluated germline genes associated with breast cancer risk among 60,000 women, half of whom were diagnosed with breast cancer and the other half unaffected [32]. Interestingly, pathogenic variants in BARD1, RAD51C, and RAD51D were associated with increased risks of estrogen receptor-negative breast cancer and triple-negative breast cancer, whereas pathogenic variants in ATM, CDH1, and CHEK2 were associated with an increased risk of estrogen receptor-positive breast cancer. Furthermore, younger women under the age of 40 were at higher risk of carrying a germline mutation linked to breast cancer. This suggests the need for more genetic counseling and improved screening assays to evaluate women at the highest risk of having a mutation, especially in underserved, minority populations. Lastly, 3% of oncologists are Black in the USA which adds to structural racism, underrepresentation of a diverse work field, and lack of trust in the minority communities [33]. Increased efforts are needed to ensure equitable access to appropriate prevention and treatment for breast cancer.

### Allostatic load impacts breast cancer health disparities

In addition to accessing care due to structural barriers, the lived experience of racism and the associated environmental challenges results in a chronic stress state that increases neural and neuroendocrine responses, known as allostatic load. This social determinant of health includes conditions in which people are born, live, grow, work, and age contributing to health-promoting vs. health-damaging resources [34]. Allostatic load is present regardless of income level and has been described in Black populations resulting in more health-damaging effects. A high allostatic load has a myriad of adverse consequences from increased frailty to more severe comorbid conditions (e.g., heart disease, anxiety, substance abuse). In breast cancer, the higher allostatic load is associated with advanced stage at diagnosis, biologically aggressive tumors, and worse quality of life [26]. Studies have noted that Black women diagnosed with breast cancer have higher allostatic loads compared to non-Hispanic White women [26]. Understanding these biopsychosocial determinants is key to improving cancer-related outcomes.

### Tumor biology contributes to survival outcomes

Tumor biology also contributes to the racial and ethnic differences in breast cancer outcomes. Breast cancer is a constellation of different molecular subtypes that vary in aggressiveness. The classic subtyping, based on estrogen receptor (ER), progesterone receptor (PR), and human epidermal growth factor receptor 2 (HER2) status, includes ER+HER2−, HER2+, and triple-negative breast cancer (TNBC). The most aggressive subtype, TNBC, occurs in 21% of Black women, but only 12% of Hispanic women and 10% of non-Hispanic White women and Asian women [25]. Age-adjusted studies have demonstrated that (younger) Black and Hispanic women have a higher incidence of hormone receptor-negative (basal-like) breast cancer than White women (Millikan Breast Cancer Research and Treatment 2008) [35–37]. Additionally, the incidence of basal-like breast cancers, often an aggressive molecular subtype of breast cancer, is lower in Japan but higher in African-American women and even more elevated in Nigerians as well as Cameroonians/Ugandan and Brazilians [32, 38–40]. Moreover, Asians who live in California are less likely to be diagnosed with TNBC than Asians living in Japan [41]. These differences may be attributed to both environmental and genetic causes. Furthermore, women with low socioeconomic status, who are more likely to identify as Black or Hispanic, are likely to be diagnosed with late-stage cancer and have increased mortality risk [42]. These breast cancer subtypes can be further classified using genomic data. The Cancer Genome Atlas (TCGA) and PAM50 analysis reveal that African-American patients have distinct genomic signatures and are more likely to have a basal subtype and TP53 mutations in which chemotherapy is the mainstay, and lower frequency of PIK3A mutations than White Americans, in which targeted therapy has been shown to improve survival [26, 43]. In a study of Hispanic Americans, six mutations accounted for 47% of hereditary mutations in a sample of patients at high-risk for breast cancer based on family history [44]. When compared to Western patients, another study demonstrated that Asian patients had higher levels of tumor-infiltrating lymphocytes, indicating differences in the immune profile of breast cancers in this population which may also lead to more targetable drugs such as immune therapy to improve outcomes [45]. These findings suggest a founder effect for groups of people descending from specific countries of origin [26], which may account for the differences in response and adverse events to therapies as well as survival outcomes among various groups.

### Conclusions

Approximately 12.7% of the US population are Black with African or Caribbean ancestry [46], yet fewer than 3% of patients enrolled in clinical trials are Black [47]. Conversely, Asian populations tend to be overrepresented in cancer clinical trials when compared to cancer incidence [26]. Diversity in patient populations in clinical research is lacking. Clinical studies predominantly draw from non-Hispanic White populations, limiting the understanding of potentially important biological and social differences that could guide treatment [47]. Within therapeutic studies, clinical trial participants poorly reflect the make-up of the US population leading to insufficient information to adequately ascertain whether the trial was reflective of the general population in terms of race and ethnicity [48]. This lack of engagement of diverse populations in clinical trials disadvantages these groups in terms of individual access to novel agents, understanding genetics, pharmacogenomics, and pharmacokinetics in specific populations and limits the oncology community’s ability to know how to optimally provide personalized treatment to these populations. Improvement in health equity in the US will require continued research in diverse populations to evaluate clinical, biological, and social factors that impact cancer.

### Competing interests

ESR reports advisory board participation from AstraZeneca, Novartis, Merck, Immunomedics, and Lilly. The other authors declare that they have no competing interests.

## Ethnicity, clinicopathologic features, and outcomes of young women with breast cancer in the UK

Alex Daly, Ramsey Cutress, Ellen Copson^*^

*Corresponding author:

Dr Ellen R Copson.

### Introduction

**Fig. 1:**
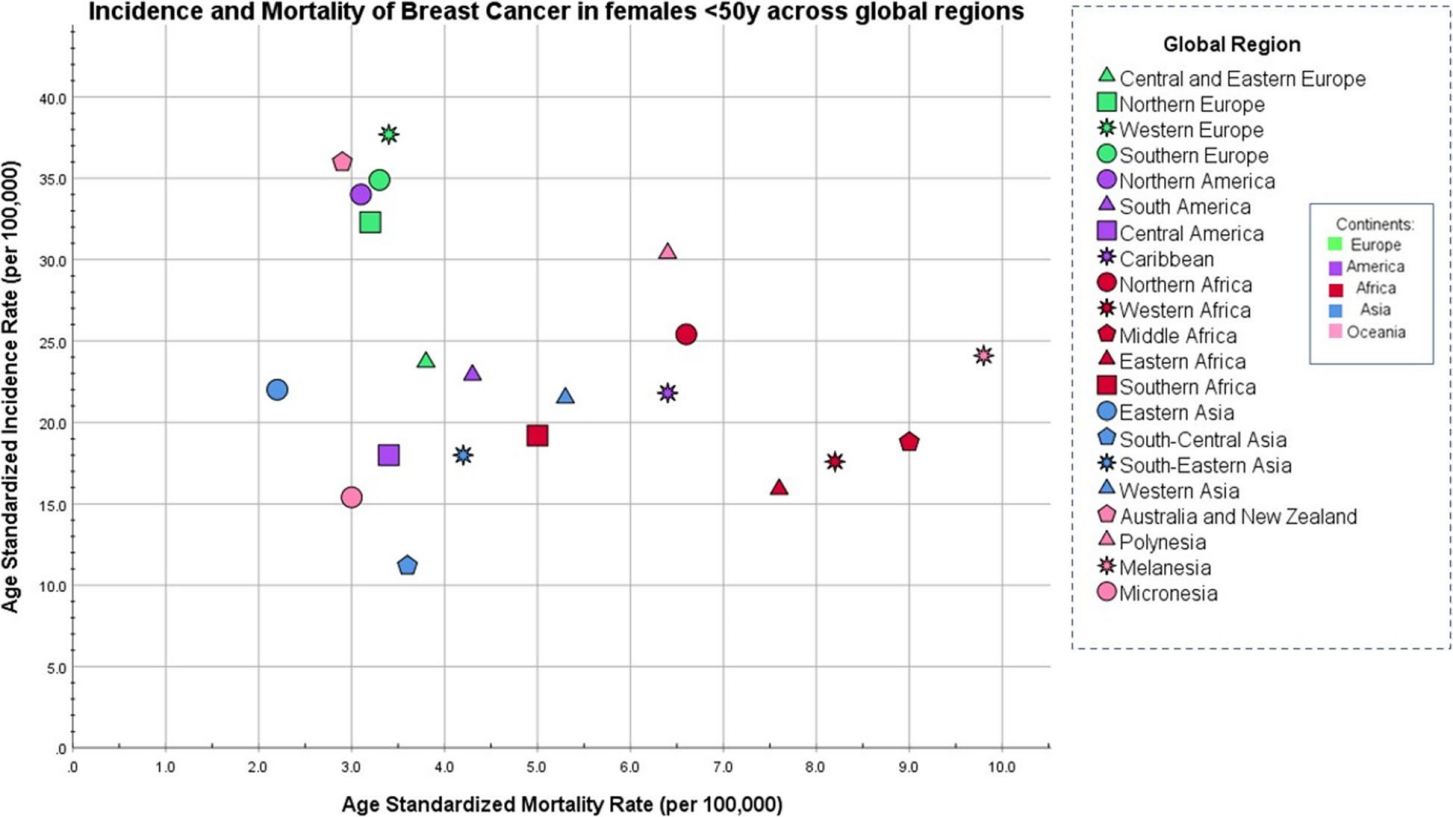
Age-standardized incidence compared with age-standardized mortality breast cancer rates across 21 regions and 5 continents in women age 0–49 years (based on publicly available data from GLOBOCAN) [49]

Breast cancer is both the most common cancer worldwide diagnosed in women aged 0–39 years and the biggest cause of cancer mortality (per year) in young women [49] accounting for 248,000 cases and 42,700 deaths each year. There are however significant differences in the incidence and survival from young-onset breast cancer between different geographical regions (Fig. 1). Most of Europe, as well as North America, Australia, and New Zealand, have a higher incidence of young-onset breast cancer with lower mortality whereas the inverse relationship is seen in many regions of Africa, Western Asia, and Melanesia (Fig. 1) [49]. While geographical trends in breast cancer incidence are attributable to breast cancer risk factor patterns and global variations in breast cancer mortality largely reflect healthcare issues, there is increasing evidence of poorer breast cancer outcomes in racial and ethnic minority women compared to other populations within the same geographical areas [50–52]. Research in this area now indicates multifactorial reasons behind inequality of outcomes encompassing variations in biology, health behavior, and socioeconomic factors [52].

### Incidence patterns and screening inequalities

Data from the UK Million Women Study, a population-based prospective cohort study of breast cancer in 1.32 million women aged 50–64 in the UK, confirmed that differences in breast cancer incidence by race and ethnicity in middle-aged women are largely explained by variations in prevalence patterns of known breast cancer risk factors including reproductive history, hormone replacement therapy, obesity, and alcohol use [53]. Although the incidence of breast cancer at any age is highest in white women (141.1 cases per 100,000) when compared to other ethnic groups (African-Americans 119.4, Asian-Americans 96.6, Hispanics 89.9 per 100,000) [54], the same trend is not seen in younger age groups. In women below the age of 50 years, the incidence rate is comparable between White and Black women and is lower in other ethnicities such as Hispanic women [54, 55]. Furthermore, the median age at diagnosis for breast cancer is lower in the Black, Hispanic, and Asian groups (Black = 56 years, Hispanic = 55 years, South Asian = 56 years) compared to White non-Hispanic women (59 years) [56]. While many factors impact the age recommendations for screening programs, the racial and ethnic composition of the population and therefore pattern of onset of breast cancer will be of relevance.

### Pathological differences between the ethnic groups

Multiple studies have reported that racial and ethnic minority women present with higher stage breast cancer than white women [50, 57, 58]. Data from our own Prospective study of Outcomes in Sporadic versus Hereditary breast cancer, a prospective cohort study of 2733 women aged 18–40 years diagnosed with primary breast cancer across 127 hospitals in the UK, confirmed that young (< 40 years) Black women had larger tumors at presentation (median tumor diameter 26 mm) than young white women (22 mm; B vs. W *p* = 0.0103) [50]. Black women were also more likely to have distant metastasis at presentation compared to Whites (B = 5.1%, W = 2.4%) [50]. In this young patient group who are not eligible for asymptomatic breast screening in the UK, such data is suggestive of delayed presentation to healthcare services despite free access to healthcare within the UK NHS. A systematic review of 18 studies by Jones et al. identified a number of factors contributing to delayed breast cancer presentation in racial and ethnic minority women including lower awareness of breast cancer symptoms and risk factors, lower priority for “breast awareness”/taboo and stigma fear of cancer diagnosis, fear of conventional treatments, mistrust of healthcare professionals, and inaccessibility of healthcare services [59]. A higher frequency of multifocal tumors in young Black (43.4%) and Asian women (37.0%) than in Whites (28.9%) may also impact patient recognition of symptoms [50].

The delayed presentation does not however explain the significant variations in breast cancer biology between ethnic groups which have been reported in multiple studies, with Black women having a higher frequency of grade 3 tumors than White and Asian women, and higher proportions of estrogen negative/progesterone negative/HER2-negative tumors than other ethnic groups [50, 52, 57]. POSH study data does not indicate statistically significant differences in *BRCA1/2* germline mutation rates to account for the increased frequency of triple-negative breast cancers in young black women compared to White women [60]. Our data does however indicate that higher body mass index (BMI) is associated with higher frequencies of young-onset triple-negative breast cancers, and the median body mass index was significantly higher in Black than white patients in the POSH study cohort [60] (Table 1).

**Table 1:** Tumor characteristics in different ethnic groups: data from the Prospective study of Outcomes in Sporadic versus Hereditary breast cancer [50]

Tumor characteristic	All^**a**^, ***n*** = 2956	White, ***n*** = 2690	Black, ***n*** = 118	Asian, ***n*** = 87	
Tumor diameter median	22.0	22.0	26.00	26.00	*W vs. B p=0.01*
Multifocal distribution	29.9%	24.64%	38.55%	32.69%	*W vs. B p = 0.004*
Nodal stage					
N0	48.9%	49.2%	43.9%	48.2%	*p = NS*
N1	51.2%	50.8%	56.1%	51.8%	
Distant metastases	2.5%	2.4%	5.1%	3.5%	*p = NS*
Grade					
1	5.7%	5.6%	0.9%	11.8%	*W vs. B p = 0.055* *W vs. A p = 0.045* *B vs. A p = 0.004*
2	33.8%	34.0%	30.0%	28.2%	
3	60.6%	60.4%	68.1%	60.0%	
Missing	2.7%	2.5%	4.2%	2.3%	
ER positive^b^	66.1%	66.5%	62.4%	57.5%	*NS*
ER negative	33.9%	33.5%	37.6%	42.5%	
Missing	0.4%	0.4%	0.9%	0.0%	
HER2 positive^b^	28.1%	28.3%	20.2%	29.7%	*NS*
HER2 negative	72.0%	71.7%	79.8%	70.3%	
Missing	13.5%	13.7%	7.6%	14.9%	
TNT^c^	19.0%	18.6%	26.1%	23.2%	*W vs. B = 0.043*
Missing	4.2%	4.2%	2.5%	5.8%	

*Abbreviations*: *ER* estrogen receptor, *HER2* human epidermal growth factor receptor 2PR ¼ progesterone receptor, *TNT* triple-negative

^a^Includes patients in an other or missing/unknown ethnic group

^b^Includes data from TMA as well as primary POSH data

^c^Includes patients with an ER-negative, HER2-negative, and PR-negative status. *p*-values obtained from the Pearson’s chi-squared test between ethnic groups and each categorical variable (excluding other ethnic groups and missing/unknown data)

### Treatment

A recent study of 164,000 UK breast cancer patients found no significant differences between the ethnic groups in the surgical management of women with early breast cancer once age and stage had been taken into consideration [61]. However, increased frequency of biologically aggressive and more advanced stage tumors result in increased treatment with mastectomy in young Black compared to young White women. This may partially explain why racial and ethnic minority patients report less favorable clinical experiences and lower satisfaction levels pertaining to their cancer treatment [62]. Reports of reduced use of hormonal therapy by racial and ethnic minority women may be explained by socioeconomic issues but require further investigation [63].

### Breast cancer survival

A number of studies have reported poorer long-term breast cancer outcomes in racial and ethnic minorities compared to White women which are not fully explained by the higher frequencies of higher stage and biologically aggressive tumors in these patient groups [50, 52, 64]. Our own data from the POSH study indicated a significantly lower 5-year overall survival of 71.1% in Black women, compared to 82.4% in White women (W vs. B: *p* = 0.0160) with a 5-year distant relapse-free survival 14.2% lower in Blacks than in Whites (W vs. B: *p* = 0.0053) [50]. Multi-variate analysis adjusting for tumor grade, stage, ER and HER2 status, and patient BMI confirmed that race and ethnicity remains an independent prognostic factor with young Black women having significantly poorer outcomes than White women (HR 1.5, *p* = 0.023). An American study of over 500,000 non-age selected breast cancer patients concluded that 37% of excess risk of death in black patients was attributable to health insurance disparities; however, even after adjusting for insurance, co-morbidities, treatment, and tumor factors, Black women had a higher risk of breast cancer mortality compared to White patients (HR 1.25 for ER-positive tumors and 1.18 for ER-negative tumors) [52]. Recent research suggests that the previously unaccounted discrepancy in outcomes could be the result of further intrinsic differences in tumor biology between ethnic groups which are not routinely recorded, including increased frequency of luminal B tumors and different mutational patterns with increased somatic *TP53* mutations [65].

## Conclusions

In summary, although there is a reduced incidence of breast cancer in Black women at all ages, the incidence in young Black women is comparable to other racial and ethnic populations and data indicate that young-onset breast cancer survival is poorer in Black women than in White populations.

There is a need to work with diverse communities to develop culturally appropriate and acceptable health promotion messages that will inform and educate about breast cancer risks and symptoms to reduce late presentations in racial and ethnic minorities while a more nuanced approach to screening services may also be required to improve quality of care for ethnic minorities. Increased recruitment of women from diverse racial and ethnic backgrounds into breast cancer clinical trials is also vital to enable further research into the additional biological and social factors contributing to breast cancer disparities which are not yet fully understood.

The WHO constitution (1946) recognizes that high-quality healthcare standards must be provided for every human being regardless of race [66]. It is of paramount importance to recognize the various challenges associated with diagnosing and managing breast cancer across racial and ethnic populations to ensure the provision of the highest quality of care across all races.

### Competing interests

EC reports honorarium from Astra-Zeneca, Novartis, Pfizer, Roche, and Lilly; advisory board participation for Pfizer, Nanostring, and Lilly; and expert panel participation for World Cancer Research Fund. RIC and EC report institutional research funding from SECA

### Acknowledgements

We acknowledge Professor Diana Eccles, the chief investigator of the POSH study, the POSH collaborators, and all the patients who participated in the POSH study. Funding for the POSH study has been provided by the Wessex Cancer Trust, Cancer Research UK (grant refs A7572, A11699, C22524); the study is a National Cancer Research Network Portfolio study.

## Impact of ethnicity on efficacy and toxicity of cyclin-dependent kinase 4/6 inhibitors in breast cancer

### Dae-Won Lee, Kyung-Hun Lee, Seock-Ah Im

Breast cancer is the most commonly diagnosed cancer globally and is the leading cause of cancer death in women [1]. Breast cancer incidence is increasing in East Asian countries, which may be due to nationwide cancer screening and lifestyle changes [67]. The peak age of breast cancer diagnosis is younger in East Asia (around age 50 years) compared to Western countries (around age 70 years) [67]. About half of patients diagnosed with breast cancer are premenopausal in East Asia. Studies show that there are molecular differences between breast cancer in Asian and Western patients [68]. Compared to Western, Asian breast cancer patients have a higher ratio of luminal B disease, a higher frequency of *TP53* mutations, and a more active immune microenvironment. While luminal A tumors generally are more indolent and sensitive to endocrine therapies, luminal B tumors have higher Ki-67 expression, lower expression of luminal-related genes, and higher frequency of *TP53* mutations. As a result, luminal B tumors have a worse prognosis and often show resistance to endocrine therapies [69].

Endocrine therapy is the main treatment option in women with hormone receptor (HR)-positive, HER2-negative advanced breast cancer. Recently, cyclin-dependent kinase (CDK) 4 and 6 inhibitors have emerged as a major milestone in these patients, showing improved progression-free survival (PFS) and overall survival (OS). In phase III pivotal randomized trials of CDK 4/6 inhibitors, Asians are underrepresented constituting 7.6–14.2% in first-line aromatase inhibitor-based trials for postmenopausal breast cancer indication and 21.0–32.0% in second-line fulvestrant-based trials or trials allowing premenopausal breast cancer [70–72] (Table 2).

**Table 2:** Asian population in pivotal CDK4/6 inhibitor trials

Trial	Line of treatment	Number of patients	Regimen	Asian population	mPFS (mo), ET+CDKi vs. ET
MONALEESA-2	1st	668	Letrozole ± ribociclib	7.6%	
MONALEESA-7	1st	672	Endocrine ± ribociclib	29.5%	30.4 vs. 11.0
MONARCH-3	1st	493	NSAI ± abemaciclib	30.0%	
PALOMA-2	1st	666	Letrozole ± palbociclib	14.2%	25.7 vs. 13.9
MONALEESA-3	1st and 2nd	726	Fulvestrant ± ribociclib	8.7%	NR vs. 12.7^a^
MONARCH 2	2nd	669	Fulvestrant ± abemaciclib	32.0%	22.8 vs. .11.6
PALOMA-3	2nd or later	521	Fulvestrant ± palbociclib	21.0%	NR vs. 5.8

*Abbreviations*: *ET* endocrine treatment, *CDKi* cyclin-dependent kinase inhibitor, *NSAI* non-steroidal aromatase inhibitor, *NR* not reached at the time of publication

^a^Pooled analysis of the MONALEESA-2, MONALEESA-3, and MONALEESA-7 trials of ribociclib (RIB) plus endocrine therapy (ET)

PALOMA-2 is a multinational trial demonstrating improved PFS with the addition of palbociclib to letrozole as first-line treatment. Ninety-five of 666 enrolled patients (14.3%) were Asian (Table 2), and they had higher incidence of neutropenia (all grade, 95.4% vs. 76.8%; grade 3/4, 89.2% vs. 62.5), leukopenia (all grade, 43.1% vs. 38.3%; grade 3/4, 32.3% vs. 23.5%), and thrombocytopenia (all grade, 27.7% vs. 13.5%; grade 3/4, 4.6% vs. 1.1%) with palbociclib compared to Western patients [73]. However, toxicities were manageable with early dose modifications, with no deterioration in the quality of life and few permanent discontinuations as a result of these events. Moreover, the PFS benefit of the addition of palbociclib was maintained in Asian patients.

PALOMA-3 trial included HR-positive advanced breast cancer patients who had relapsed or progressed during or after prior endocrine therapy. Twenty percent of enrolled patients were Asian (Table 2) and PFS improvement by palbociclib was similar in Asians compared to non-Asians (HR 0.485 and 0.451, respectively) [74]. In the palbociclib arm, Asian patients had lower incidence of fatigue (19% vs. 44%) and had higher incidence of neutropenia (92% vs. 78%), stomatitis (41% vs. 24%), rash (32% vs. 11%), and nasopharyngitis (21% vs. 10%).

In the post hoc analysis of MONARCH2 (fulvestrant ± abemaciclib) and MONARCH3 (nonsteroidal aromatase inhibitor ± abemaciclib), the efficacy of abemaciclib in East Asians was consistent with the ITT populations [75]. In the MONARCH2 trial, East Asian patients who were treated with fulvestrant plus abemaciclib had a higher incidence of neutropenia (all grade, 67.8% vs. 35.3%; grade ≥ 3, 44.5% vs. 17.6%) and ALT elevation (all grade, 23.3% vs. 8.5%; data not reported for grade ≥ 3) compared to non-East Asian ones [75]. Diarrhea was frequently found in both East Asians and non-East Asians (all grade, 90.4% vs. 84.4%; grade ≥ 3, 14.4% vs. 12.9%). In the MONARCH3 trial, all grade neutropenia (57.8% vs. 37.3%) and ALT elevation (all grade, 32.4% vs. 10.7%; grade ≥ 3, 13.7% vs. 3.1%) was more frequently observed in East Asian, but there was no difference in neutropenia grade ≥ 3 (29.4% vs. 21.3%) and diarrhea (all grade, 88.2% vs. 79.6%). In the pooled analysis of efficacy and safety in Asian patients in the MONALEESA-2, MONALEESA-3, and MONALEESA-7 trials of ribociclib plus endocrine therapy, the most common grade 3/4 adverse event (AE) neutropenia was similar in Asian (47.1%) and non-Asian (45.6%) RIB-treated patients [76].

In a meta-analysis of 4 phase III trials (MONALEESA-2, MONALEESA-7, PALOMA-2, MONARCH3) of first line CDK4/6 inhibitor plus endocrine therapy, 19.7% of enrolled patients were Asians (492/2499) [70]. The hazard ratio of adding CDK4/6 inhibitor in terms of PFS was 0.39 (95% CI 0.29–0.51) for Asian and 0.62 (95% CI 0.54–0.71) for non-Asians (*p* = 0.002, treatment-ethnicity interaction) in first line trials. Toxicity data by ethnic subgroup was available from 2 studies in this meta-analysis (MONALEESA-2 and PALOMA-2). In the CDK4/6 inhibitor arm, Asian had higher incidence of neutropenia (90.9% vs. 75.1%, *p <* 0.001) and lower incidence of diarrhea (15.2% vs. 32.1%, *p* = 0.003) compared to non-Asian population [70]. In addition, dose reduction due to adverse events (58.0% vs. 40.0%, *p* < 0.001) and drug interruptions (75.0% vs. 66.2%, *p* = 0.05) were also more frequent in Asians compared to non-Asians. In the MONALEESA-2 (letrozole ± ribociclib), MONALEESA-3 (fulvestrant ± ribociclib), and MONALEESA-7 (nonsteroidal aromatase inhibitor ± ribociclib) trials, dose reduction in Asians were done in 57% (50% in non-Asians), 57% (36% in non-Asians), and 45% (32% in non-Asians), respectively [77]. Dose reduction rates of abemaciclib in East Asians were 51.4% in the MONARCH2 trial (42.9% in the ITT) and 46.1% in the MONARCH3 trial (43.4% in the ITT) [75]. In the PALOMA-2 and PALOMA-3 trials, Asians had higher incidence of dose reduction (56.9% vs. 32.5% and 52% vs. 29%, respectively) [73, 74]. In addition, Asians had more cycle delays (51% vs. 32%) and lower median relative dose (87% vs. 98%) of palbociclib compared to non-Asians in the PALOMA-3 trial [74].

Collectively, CDK4/6 inhibitors show comparable efficacy but may have higher toxicity in the Asian population compared to the non-Asian population. In the PALOMA-2 trial, the mean steady-state trough concentration (C_trough_) of palbociclib was higher in Asians relative to non-Asians (93.8 and 61.7 ng/mL, respectively), and clearance was lower compared with non-Asians (33.6 ± 10.6 vs. 48.0 ± 18.2 L/h) [73]. There was no relationship between C_trough_ and body dimensions in this population [73]. In contrast, in the PALOMA-3 trial, the mean steady-state C_trough_ of palbociclib was similar between Asians and non-Asians (85.7 and 74.8 ng/mL, respectively) [74]. In the pharmacokinetic analysis of the MONARCH2 and MONARCH3 trials, race was not a significant covariate on any of the PK parameters [75]. CYP3A4, P-glycoprotein (P-gp), and breast cancer resistance protein (BCRP) are involved in the pharmacokinetics of CKD4/6 inhibitors [69], and CYP3A4 activity may be different according to the ethnicity [78, 79]. These factors might have affected different toxicity profiles of CDK 4/6 inhibitors in Asians. In addition, Asians had low baseline absolute neutrophil count (ANC) in patients enrolled in the PALOMA-3 trial (19% lower) [74]. In-depth pharmacogenetic research could provide explanations for these differences in the near future.

### Competing interests

SAI is a recipient of research funds from AstraZeneca Inc., Roche, and Pfizer and has consultant and advisory roles for Amen, AstraZeneca, Eisai, Hanmi Corp., Lilly, Novartis, Pfizer, and Roche. The other authors declare that they have no competing interests.

## Ethnic differences in *BRCA* mutant breast cancer and PARP inhibitors

### Yeon Hee Park

Breast cancer is the most female prevalent cancer in Korea, and the highest incidence was reported in the 40–49 year age group [80]. Furthermore, the incidence of breast cancer is increasing in Asia, including in Korea. As the incidence of breast cancer increased, breast cancer mortality rates are also increasing. This resulted in breast cancer representing the leading cause of death in women aged 40 to 59 and the third leading cause of death in women aged 30–39. Because of epidemiological differences in breast cancers in Asia compared with Western countries [81], 15% are younger than 40 years and 55% are younger than 50 when they are diagnosed with breast cancer in Asia [67].

These differences in epidemiology may be related to the prevalence of hereditary breast cancer (HBC) according to ethnic backgrounds. There is an age gap between Western and Asian breast cancer patients. About half of the patients in Asia are premenopausal when they are diagnosed as breast cancer. However, the overall prevalence of *BRCA 1* and/or *2* mutations in breast cancer is not well defined. According to Winter et al. [82], it has been estimated that approximately 7% of breast cancers are associated with germline *BRCA 1* and/or *2* mutation and additional 3% have somatic *BRCA 1* and/or *2* mutation. However, founder mutations in certain geographical locations do skew these data. While *BRCA 1* and/or *2* mutations are widely associated with triple-negative breast cancer (TNBC), the clinical community is less likely to assess *BRCA 1* and/or *2* mutations in hormone receptor (HR)-positive disease. However, evidence suggests that HR-positive patients account for at least half of all *BRCA 1* and/or *2* mutation carriers: 1 in 17 HR-positive patients have a germline *BRCA 1* and/or *2* mutations (65% of breast cancer germline *BRCA 1* and/or *2* mutation population), the majority of these would be germline *BRCA2* mutations [83, 84]. One in 6 TNBC patients have a germline *BRCA 1* and/or *2* mutation (30% of breast cancer germline *BRCA 1* and/or *2* population) [82], the majority of these would be germline *BRCA1* mutations [85]. According to the OlympiAD study, 121 of the 895 (13.5%) Asian patients screened for the study using a Myriad test were found to have a germline *BRCA 1* and/or *2* mutation, which is a slightly higher mutation rate than observed in the other populations in the study [86].

We elucidated clinical characteristic and genomic analyses in germline *BRCA1* or *BRCA2* mutated breast cancer in Korean women. Among 2720 total breast cancer cases tested for germline *BRCA* mutations at Samsung Medical Center (SMC), 386 (14.2%) showed either pathogenic germline *BRCA 1* or *2*, or both mutations [87]. This screening test is reimbursed for patients with risk factors for HBC in Korea. This guideline ironically has a more narrow disease spectrum compared with National Comprehensive Cancer Network (NCCN) guidelines. Some of the patients who conducted targeted sequencing and whole transcriptomic sequencing (WTS) at the same time were also analyzed in this study. Median tumor mutation burden (TMB) was 6.53/megabase (MB) in germline *BRCA1* and 6.44/MB in germline *BRCA2* mutations. Through the PAM50 results, 31% of ER-positive patients were re-classified to basal type: 21% were ER-positive in germline *BRCA1* mutation and 80% in germline *BRCA2* mutations. When we observed somatic mutations through targeted sequencing, accompanying *TP53* mutations were reported in 62% of the patients. However, *BRCA2* mutation was exclusive with *TP53* mutations, and co-occurrence was seen in *BRCA1* and *TP53* mutations [87].

A study on Korean breast cancer patients reported germline *BRCA 1* and/or *2* mutations in 13.1% of overall patients and 14.5% of patients with less than 60 years in unselected TNBC patients [88]. Importantly, there has been a lot of effort to identify clinically significant pathogenic mutation sites in Korean breast cancer patients. From these studies, the *BRCA1* L1780P site could be reclassified as a pathogenic mutation in the ACMG guideline [89–91].

It seems that *BRCA* mutation prevalence is approximately 10–15% in the non-high-risk HR-positive, non-selected population in Korea. This relatively higher prevalence of BRCA mutations in the HR-positive population may be due to higher ER-positive breast cancer prevalence among young breast cancer patients in Asians compared with US populations [67]. These ethnic differences in *BRCA* mutations, especially for patients with ER-positive breast cancer between west and east, should be validated through epidemiological evaluation. This may also affect the positioning of PARP1 inhibitors, which has been approved by the Food and Drug Administration (FDA).

In summary, there is interethnic heterogeneity of HBCs and a complex interplay between environmental and intrinsic factors. There are ethnic differences in the prevalence of germline *BRCA 1* and/or *2* mutations. However, the actual prevalence of germline *BRCA 1* and/or *2* mutations remains to be defined. Furthermore, there are ethnic differences in pathogenic mutation sites. Founder mutations could be different according to the geographic area. Re-positioning of PARP1 inhibitors for patients with breast cancers may be needed, especially for ER-positive breast cancers. Extension from germline *BRCA 1* and/or *2* mutation to homologous recombination deficiency (HRD) signature would be defined.

### Competing interests

SHP reports consultancy/advisory role for AstraZeneca, Pfizer, Eisai, Roche, Daiichi-Sankyo, Eli Lilly, and Novartis Pharmaceuticals; patents and royalties from Hanmi; honoraria from AstraZeneca, Pfizer, Eisai, Roche, Daiichi-Sankyo, and Novartis; and grant/research funding from AstraZeneca, Merck, Pfizer, Novartis, Alteogen, and Roche.
